# HER2-low breast cancer is immune-cold: insights into tumor-infiltrating immune cells and implications for immunotherapy

**DOI:** 10.1038/s41523-025-00867-z

**Published:** 2025-12-03

**Authors:** S. Pizzamiglio, A. Blanda, V. Appierto, P. Minicozzi, M. G. Carnevale, M. C. De Santis, B. Re, L. Cortesi, E. Gasparini, G. Arpino, M. Giuliano, V. Molinaro, M. V. Iorio, L. De Cecco, A. Bertolotti, S. Brich, Andrea Vingiani, C. De Marco, G. Pruneri, P. Verderio, S. Di Cosimo

**Affiliations:** 1 Department of Epidemiology and Data Science, Fondazione IRCCS Istituto Nazionale dei Tumori, Milan, Italy; 2 Department of Advanced Diagnostics, Fondazione IRCCS Istituto Nazionale dei Tumori, Milan, Italy; 3 Department of Radiation Oncology, Fondazione IRCCS Istituto Nazionale dei Tumori, Milan, Italy; 4 Breast Unit, Fondazione IRCCS Istituto Nazionale dei Tumori, Milan, Italy; 5 Pharmacy, Fondazione IRCCS Istituto Nazionale dei Tumori, Milan, Italy; 6 Medical Oncology Unit, Azienda Unità Sanitaria Locale-IRCCS, Reggio Emilia, Italy; 7 Department of Clinical Medicine and Surgery, University Federico II, Naples, Italy; 8 Department of Experimental Oncology, Fondazione IRCCS Istituto Nazionale dei Tumori, Milan, Italy; 9 Department of Oncology and Hemato-Oncology, University of Milan, Milan, Italy

**Keywords:** Cancer, Immunology, Oncology

## Abstract

This study investigated, for the first time, the association between HER2 expression, immune infiltration inferred by CIBERSORTx, and immunotherapy response in HER2-negative early breast cancer (EBC). Gene expression was analyzed in prospective discovery (*n* = 104), confirmatory (*n* = 81), and independent (ABiM, *n* = 318) cohorts. HER2 expression was measured using a 20-gene signature yielding progressively higher scores from HER2-0 to HER2-low, as routinely defined. Increased HER2 expression was consistently associated with reduced immune-infiltration, especially cytotoxic (CD8^+^) T cells and M1 macrophages; and hormone receptor (HR)-specific depletions with significant interactions for mast cells resting and neutrophils. In analysis of covariance, HER2 expression independently predicted low B-naïve and plasma cell abundance. In a neoadjuvant immunotherapy real-world cohort (*n* = 111), HER2-low primary tumors had numerically lower midcourse (28% vs. 44%) and pathological complete response (64% vs. 72%) compared to HER2-0. These findings show that HER2 expression defines immune-cold HER2-negative EBC, hindering immunotherapy and supporting HER2-targeted combination in HER2-low EBC patients.

## Introduction

Breast cancer affects over two million people annually and represents the leading cause of cancer-related death among women worldwide^1^. Prognosis and treatment decisions primarily rely on disease staging and on molecular subtypes identified through surrogate markers^2,3^. According to immunohistochemical evaluation (IHC) of hormone receptors (HR) and human epidermal growth factor receptor 2 (HER2), breast cancer is classified as estrogen (ER) and/or progesterone receptor (PgR) positive (65%), HER2-positive (20%), and triple-negative (TNBC, 15%)^3^. More recently, HER2-low tumors defined as IHC 1+ or IHC 2+ without HER2 gene (*ERBB*2) amplification have emerged as a new category of clinical interest, following the therapeutic success of HER2-targeted antibody drug conjugates (ADCs)^4^.

Breast cancer subtypes are intrinsically heterogeneous not only in their gene expression characteristics but also in their repertoire of tumor-associated and specific antigens, which shape the composition of microenvironment^5^. Many studies have shown that higher levels of tumor-infiltrating lymphocytes (TILs) are associated with favorable prognosis in TNBC and HER2-positive breast cancer^6^. By contrast, HR-positive tumors typically show fewer TILs, which are associated with poor prognosis^7^, underscoring that the composition of the immune infiltration is crucial for immune response. More detailed analyses have reported that intra-tumoral helper (CD4⁺) T and cytotoxic (CD8⁺) T cells generally confer favorable outcomes, whereas regulatory T cells, and myeloid-derived suppressor cells facilitate immune evasion and are associated with poor outcome^8^.

Within this context, the immune landscape of HER2-low breast cancer remains particularly elusive. Preliminary evidence suggests that HER2-low may harbor lower infiltration of cytotoxic (CD8⁺) T cells and natural killer (NK) compared to other subtypes^9^, while transcriptomic studies indicate that immune differences may be more closely associated with ER status than with HER2 expression alone^10^. This uncertainty highlights the need for refined investigations on the interplay between HER2 expression levels and the immune microenvironment.

A key challenge in advancing this understanding lies in the inherent difficulty of accurately assessing HER2-low expression by IHC, which is semi-quantitative, prone to inter-observer variability and technical inconsistencies, ultimately limiting precise patient stratification^11^. To overcome this, we recently published a 20-gene expression-based classifier that allows continuous quantification of HER2 expression levels, with values increasing progressively from HER2-0 onward^12^. This signature, functionally enriched for genes involved in lipid and steroid metabolism, immune response, and peptidase regulation, displays a characteristic bell-shaped distribution across IHC categories, peaking in HER2 IHC 1+ and 2+ and showing significantly lower values in 0 and 3+ tumors, outperforming *ERBB2* mRNA levels in distinguishing HER2-low^12^. Herein, we aimed to characterize the immune infiltration of HER2-negative early breast cancer according to HER2 expression by the 20-gene classifier and IHC categories, to explore associations with specific tumor-infiltrating immune cell (TIIC) populations overall and by HR status, and to evaluate implications for response to treatment.

## Materials and methods

This study was designed to investigate the association between HER2 expression, TIICs by gene expression profiling (GEP), and response to immunotherapy. A total of 614 patients were analyzed.

Gene expression was profiled in three cohorts of women with HER2-negative early breast cancer: a prospective discovery (*n* = 104) and a confirmatory cohort (*n* = 81), both treated at Fondazione IRCCS Istituto Nazionale dei Tumori, Milan; and an independent external cohort from the ABiM study (*n* = 318)^13,14^. Available clinical and pathological data included patient age, tumor size, grade, nodal status, HR and HER2 status as assessed by routine diagnostic testing.

Response to immunotherapy was analyzed in a separate cohort of patients treated with pembrolizumab-based neoadjuvant chemotherapy^15^ (*n* = 111) in three oncology centers: Fondazione IRCCS Istituto Nazionale dei Tumori, Milan, Azienda Ospedaliero-Universitaria Policlinico di Modena-Reggio Emilia, and Federico II University, Naples.

### Gene expression analysis

GEP was obtained from FFPE tumor tissue using the Affymetrix U133 Plus 2.0 platform in the discovery cohort; and from frozen tissue using the Illumina Human HT-12_V3.0 platform in the confirmatory cohort. GEP from the ABiM cohort was downloaded from the website https://www.ncbi.nlm.nih.gov/geo/. Normalized GEP data were used to assess HER2 expression continuously using the previously published 20-gene expression classifier^12^; the *ERBB2* and *ESR1* levels; the total TIL gene signature scores^16^; and to compute the absolute proportion of TIICs by CIBERSORTx^17,18^ using the leukocyte gene signature matrix LM22 to estimate the proportion of 22 immune cells: B cells naïve and B cells memory; plasma cells; 7 types of T cells (CD8, CD4 naïve, CD4 memory activated, follicular helper, regulatory, γδ); NK cells resting; NK cells activated; monocytes; macrophages M0, M1, and M2; dendric cells (DC) resting; mast cells resting; mast cells activated; neutrophils; eosinophils and DC activated (https://cibersortx.stanford.edu/). TIIC abundance was analyzed in continuum. However, for TIICs with 25% to 75% of values equal to zero, data were dichotomized as 0 (absence) versus (vs) >0 (presence). TIICs with more than 75% of zero values were excluded from statistical analysis.

### Treatment response

Pathological complete response (pCR) was defined as the absence of residual invasive disease in both the breast (ypT0 or ypTis) and axillary lymph nodes (ypN0). Complete response (CR) was assessed after 4 cycles of carboplatin, paclitaxel, pembrolizumab as the absence of detectable tumor in the breast and axillary lymph nodes based on either mammography or ultrasound. Both pCR and CR were analyzed according to HER2 expression by IHC, as HER-0 vs. HER2-low (HER2 1+ and 2 + ).

### Statistical analysis

Spearman’s rank correlation coefficients (ρ) and related 95% confidence intervals (95%CI) were estimated to assess the association between HER2 expression (20 gene signature) and continuous TIICs, overall and in HR-positive and HR-negative subgroups. Wilcoxon test p-values were provided for categorical TIICs. Differences in continuous and categorical TIICs between HR-positive and HR-negative subgroups were assessed by Wilcoxon and chi-squared tests, respectively. Bonferroni correction was applied to adjust the resulting p-values for multiple comparisons.

Univariate logistic regression models^19^ were used to estimate the odds of detecting TIIC high (vs. low) abundance in relation to a 1-standard deviation increase in HER2 expression (20 gene signature). For this purpose, continuous TIIC variables were dichotomized based on their median value: values equal to or above the median were defined as *high abundance*; and, values below the median as *low abundance*. Odds ratios (ORs) and corresponding 95%CI were reported separately for HR-positive and HR-negative subgroups. Additionally, logistic regression models including HER2 expression (20 gene signature), HR status and their first-order interaction were performed. To investigate the association between clinical variables and TIIC abundance while accounting for HR status, logistic regression models were fitted for dichotomized age (≤50 vs >50 years), tumor grade (I-II vs III), tumor size (≤2 cm vs > 2 cm), including TIIC abundance, HR status and their first-order interaction. Two-way ANCOVA models including HR status, HER2 IHC category, and HER2 expression and all their first-order interactions were used to estimate the marginal effect of HER2 expression on each analysed TIIC. Univariate logistic regression models were used to assess the odds of achieving a radiological complete response or a pCR (vs. partial or no response) to immunotherapy. All statistical analyses were done using SAS Studio (version 5.2; SAS Institute, Inc., Cary, NC, USA) and RStudio (version 4.4.2), with a nominal alpha level of 5%.

### Ethical approval

This study was conducted in accordance with the Italian Data Protection Authority’s guidance for IRCCSs, public or private institutes that integrate advanced patient care with biomedical research. Pursuant to Article 110-bis, paragraph 4, of the Italian Privacy Code, health data initially collected for care purposes may be used for authorized research by IRCCSs without requiring separate consent. Written informed consent was obtained from all patients in GEP analysis at study entry and covered future biomarker research^12^. The study complied with the Declaration of Helsinki.

## Results

### Study patients

Clinical and pathological characteristics of patients included in the GEP analysis are summarized in Table 1. In the discovery cohort, median age was 49 years (41–55), with most tumors measuring 2–5 cm (83%), grade II (65%), HR-positive (79%), and predominantly HER2 IHC 1+ (58%). In the confirmatory cohort, median age was 61 years (48–73), with 48% of tumors measuring ≤2 cm, a balanced distribution of tumor grades, and 37% of cases showing HER2 IHC 2 + . Across cohorts, most tumors were luminal by PAM50 subtypes, followed by basal-like and HER2-enriched.

**Table 1:** Patient and primary tumor characteristics

	Discovery cohort *n* = 104	Confirmatory cohort *n* = 81
Age (median, range), years	49 (41–55)	61 (48–73)
Tumor size, *n* (%)		
≤ 2 cm	14 (13.4%)	39 (48.4%)
2–5 cm	86 (82.7%)	38 (46.9%)
> 5 cm	3 (2.9%)	3 (3.7%)
*missing*	1 (1%)	1 (1%)
Grade, *n* (%)		
I		
II	68 (65.4%)	40 (49.4%)
III	36 (34.6%)	41 (50.6%)
Hormone receptor, *n* (%)		
Positive	82 (78.8%)	68 (83.9%)
Negative	22 (21.2%)	13 (16.1%)
HER2 IHC, *n* (%)		
0	32 (30.8%)	25 (30.9%)
1+	60 (57.7%)	26 (32.1%)
2+	12 (11.5%)	30 (37.0%)
PAM50 subtype, *n* (%)		
Luminal A	38 (36.5%)	45 (55.5%)
Luminal B	21 (20.2%)	14 (17.3%)
HER2-enriched	8 (7.7%)	2 (2.5%)
Basal-like	18 (17.3%)	15 (18.5%)
Normal-like	19 (18.3%)	0
*undetermined*	0	5 (6.2%)

### Immune cell distribution according to hormone receptor status

Out of the 22 predefined TIIC populations estimated by CIBERSORTx, 14, 16, and 15 showed non-zero values in more than 25% of samples in the discovery, confirmatory, and ABiM cohorts, respectively, and were thus included in the analysis. TIICs were analyzed overall and by HR status. M2, M0 macrophages, and T cells CD8, were the most abundant immune populations overall. As expected, M0 and M1 macrophages, and T cells CD8, were more abundant in HR-negative compared to HR-positive tumors (Bonferroni-adjusted *p* < 0.05) (Supplementary Fig. 1).

### HER2 and immune cell abundance association

**Fig. 1:**
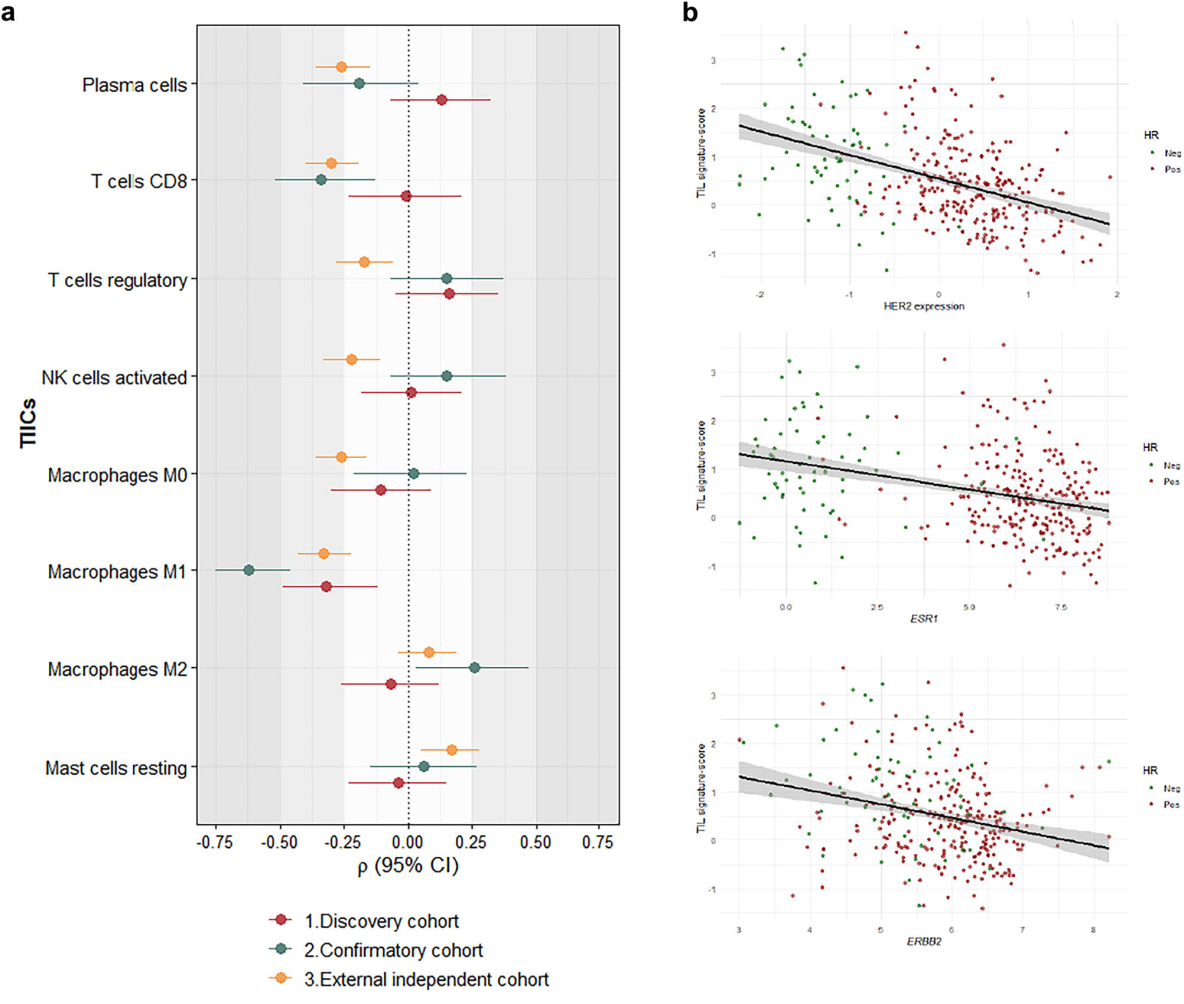
Associations of HER2 20-gene signature, ESR1 and ERBB2 expression with TIICs and TILs across study cohorts. **a** Spearman’s rank correlations between HER2 (20-gene signature) and tumor-infiltrating immune cell (TIIC) abundance in study cohorts. The ρ coefficient reflects the strength and direction of correlation between ranked variables: values close to ±1 indicate strong association, and a negative sign (−) denotes an inverse correlation, i.e., higher HER2 expression (20-gene signature) is associated with lower immune cell abundance. Correlation strength was interpreted based on absolute ρ values: weak (0.1–0.3), moderate (0.3–0.5), and strong (>0.5). In the figure, these categories are visually represented by progressively darker shaded grey bands, corresponding to increasing correlation strength. Horizontal bars represent 95% confidence intervals (95% CI). **b** Association between TILs and HER2 expression (20-gene signature), *ESR1*, and *ERBB2*. Scatter plots with linear regression lines showing the association between total tumor-infiltrating lymphocytes (TILs) as assessed by the Danaher signature scores, and expression of HER2 (20-gene signature), *ESR1*, and *ERBB2* in the ABiM cohort (*n* = 318). Each dot represents a tumor sample, color-coded by hormone receptor (HR) status (red, HR-positive; green, HR-negative). Shaded areas represent 95% confidence intervals of the regression lines.

HER2 expression as assessed by the 20-gene signature showed a moderate to strong inverse correlation (ρ = −0.3 to −0.6) with T cells CD8 and M1 macrophages, indicating that higher levels were associated with lower infiltration of these cytotoxic and pro-inflammatory immune populations (Fig. 1, panel *a*; Supplementary Table 1). These associations were consistent across cohorts reflecting a reproducible pattern. Notably, the inverse correlation between total TILs and HER2 20-gene signature (ρ = −0.44, 95%CI −0.53 to −0.34) was stronger than that observed with either single receptor expression, i.e., *ESR1* (ρ = −0.34, 95%CI −0.44 to −0.23), *ERBB2* (ρ = −0.28, 95%CI −0.38 to −0.17)(Fig. 1, panel b).

### Differential immune cells by HER2 expression within HR subgroups

**Fig. 2:**
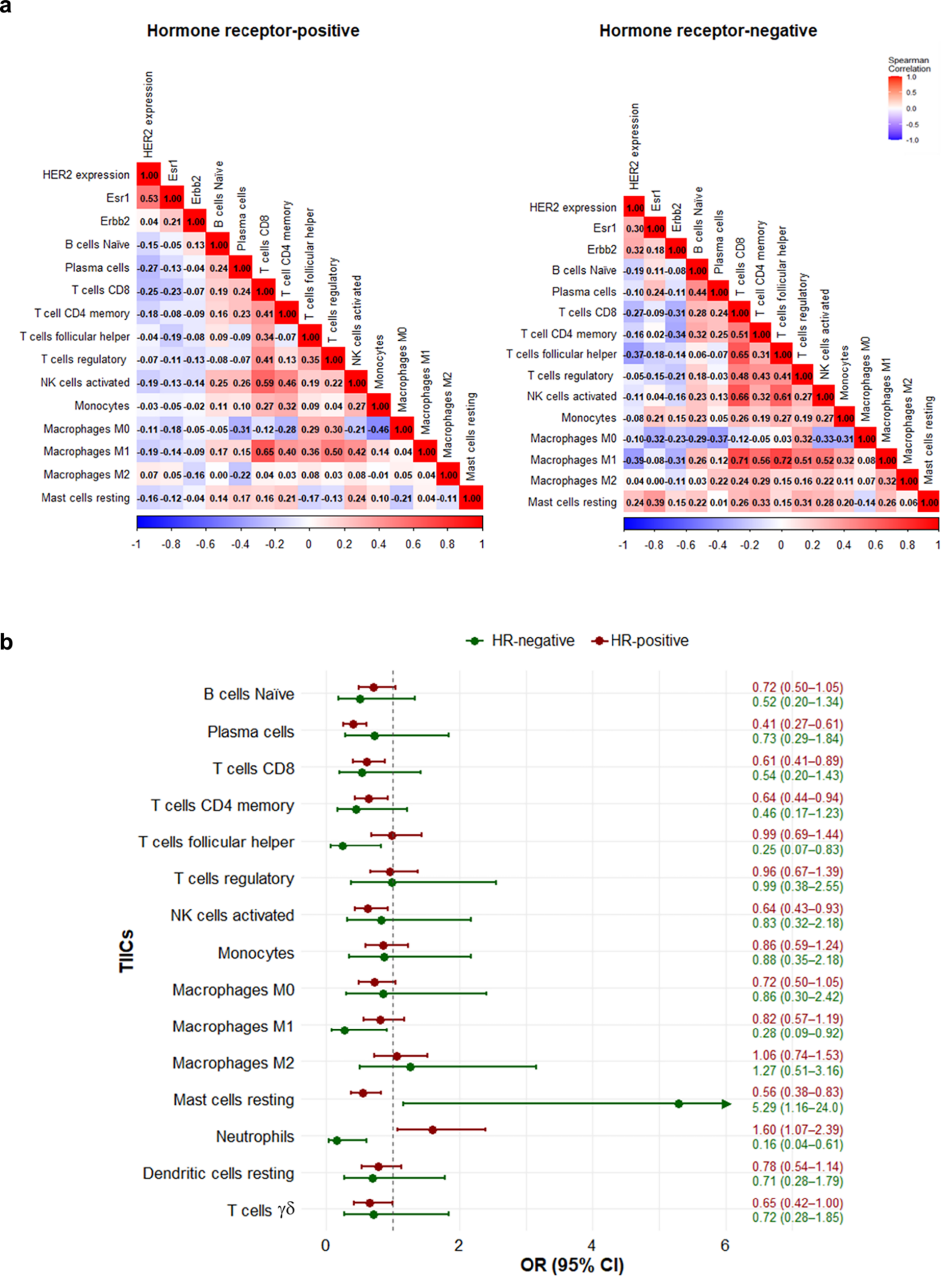
HR status–stratified analysis of the impact of HER2 expression on TIICs. **a** Correlation matrix of HER2 (20-gene), *ERBB2*, *ESR1*, and immune cell populations stratified by hormone receptor status. Spearman correlation matrices showing pairwise associations between HER2 expression (20-gene signature), *ERBB2*, *ESR1*, and the abundance of tumor-infiltrating immune cells (TIICs), stratified by hormone receptor (HR) status: HR-positive (left) and HR-negative (right). Each cell displays the Spearman’s ρ coefficient, with color intensity representing the strength and direction of the correlation (red= positive, blue= negative). Stronger associations appear in darker shades. **b** Immune cell depletion associated with HER2 (20-gene) expression according to hormone receptor status Forest plot showing odds ratios (ORs) and 95% confidence intervals (95% CI) for lower abundance of dichotomized tumor-infiltrating immune cells (TIICs) per one standard deviation increase in HER2 expression assessed by a 20-gene signature, stratified by hormone receptor (HR) status. Green markers represent HR-negative tumors and red markers HR-positive tumors. OR < 1 indicates a negative association (immune depletion). Notably, mast cells resting and neutrophils show significant HER2-HR interaction.

In HR-positive, HER2 expression was inversely correlated with moderate strength with plasma cells (ρ = −0.27; 95%CI −0.38, −0.15), T cells CD8 (ρ = −0.25, −0.37; −0.12). Weaker inverse correlations were observed with B cells naïve (ρ = −0.15; −0.28, −0.03), NK cells activated (ρ = −0.19; −0.31, −0), macrophages M0 (ρ = −0.11; −0.23, −0.001) and M1 (ρ = −0.19; −0.30, −0.06). In HR-negative tumors, the correlations were less pronounced for T cells CD8 and NK cells activated, whereas macrophages M1 retained a significant moderate inverse correlation (ρ = −0.39, 95%CI −0.59; −0.15) (Supplementary Table 2, Fig. 2, panel *a*).

### Multivariable analysis of HER2 expression and immune cell abundance

Moving beyond pairwise relationships between HR status and categorical TIICs, we next quantitatively assessed how incremental HER2 expression by the 20-gene signature impact the abundance of TIIC populations (Fig. 2, panel *b*). Specifically, in HR-positive tumors, 1-standard deviation increase in HER2 20-gene signature levels was associated with a 40% to 60% reduction in the odds of harboring plasma cells (OR 0.41, 95%CI 0.27–0.61), T cells CD8 (0.61, 0.42–0.89), T cells CD4 memory (0.64, 0.44–0.94), NK cells activated (0.64, 0.43–0.93), and T cells γδ (0.65, 0.42–1.00). In HR-negative tumors, HER2 increased expression was significantly associated with reduced odds of T cells follicular helper (0.25, 0.07–0.83) and macrophages M1 (0.28, 0.09–0.92). Notably, in HR-positive tumors, HER2 expression levels were associated with depletion of mast cells resting (0.56, 0.38–0.83) and enrichment of neutrophils (1.60, 1.07–2.39). In contrast, in HR-negative tumors, this trend was opposite for both mast cell (5.29, 1.16–24.0; p _interaction_ = 0.005) and neutrophils (0.16, 0.04–0.61; p _interaction_ = 0.001) (Fig. 2, panel *b*).

**Fig. 3:**
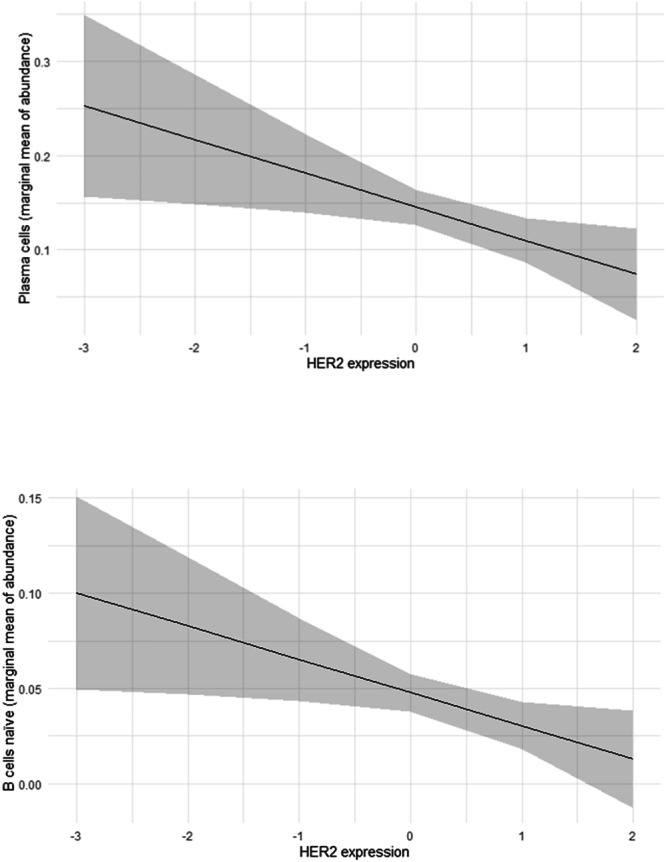
Marginal effect of HER2 expression on the abundance of plasma cells and B cells naïve. Line plot showing the marginal mean abundance of plasma cells and naïve B cells as a function of HER2 expression (20-gene signature), derived from the analysis of covariance adjusted for hormone receptor (HR) status, HER2 IHC category, and their first-order interaction. The black line represents the fitted trend, and the grey shaded area indicates the 95% confidence interval. The downward slope reflects an inverse association: as HER2 expression increases, immune cell abundance decreases, consistent with an immune-cold phenotype.

To further explore these findings, we implemented two-way ANCOVA models to assess the effect of HER2 expression by the 20-gene signature on TIIC abundance, adjusting for HR status, HER2 IHC, and their interactions. As shown in Fig. 3, higher HER2 expression was significantly associated with lower mean levels of naïve B cells and plasma cells, after adjustment for HR status and HER2 IHC, indicating a consistent depletion of humoral immune components. This marginal effect on naïve B and plasma cells remained significant after further adjustment for age, tumor size, and the interaction between HR status and TIIC abundance. No additional significant marginal effects were observed.

Impact of HER2 on response to neoadjuvant immunotherapy Midcourse and pathological response to neoadjuvant paclitaxel, carboplatin, and pembrolizumab were evaluable in 71 (64%) and 86 (77%) patients, respectively. Compared to patients with HER2-0 tumors, those with HER2-low tumors showed numerically lower CR (28% vs. 44%; OR 0.51, 95% CI 0.19–1.37) and pCR (64% vs. 70%; OR 0.67, 95% CI 0.28–1.74) (Table 2).

**Table 2:** Response to neoadjuvant pemobrolizumab in HER-0 and HER2-low patients

	Patients		HER2 by IHC			
			0		1+ or 2 +	
	*n*	%	*n*	%	*n*	%
**Midcourse response**	71					
complete	26	37	17	44	9	28
partial	45	63	22	56	23	72
**Odds Ratio (95% CI)**			1	(reference)	0.506	(0.19–1.37)
**Pathological complete response**	86					
yes	59	69	36	72	23	64
no	27	31	14	28	13	36
**Odds Ratio (95% CI)**			1	(reference)	0.688	(0.28–1.74)

## Discussion

This is the first study to analyze tumor-infiltrating immune cells and their association with HER2 expression in HER2-negative early breast cancer. According to our analysis, increased HER2 levels as assessed by a 20-gene signature were broadly associated to reduced immune cell infiltration, with distinct nuances depending on HR status, and independently predicted depletion of naïve B and plasma cells. Collectively, these findings suggest that HER2-low breast cancer is characterized by an immune-cold microenvironment.

Data on immune infiltration in HER2-low breast cancer are still limited and mostly recent. Prior studies have shown that HER2-low tumors present fewer TILs than HER2-positive and HER2-0 tumors^209^. This holds true regardless of HR status^21^. In TNBC, where TILs are recognized as a favorable prognostic marker^22^, this depletion has been proposed to explain the poorer outcomes associated with low HER2 expression^21^. Our data not only confirm these findings, showing a consistent inverse correlation between HER2 levels and TIL scores, but further indicate that cytotoxic (CD8^+^) T cells and M1 macrophages are the most markedly reduced immune populations. Cytotoxic (CD8^+^) T cells exert antitumor effects by recognizing tumor antigens and releasing cytotoxic cytokines^23^, and their infiltration is consistently associated with improved survival in HER2-positive and TNBC patients^24^. Tumor-associated macrophages are heterogeneous, as M1 promote Th1-type antitumor immunity, while M2 facilitate tumor progression and immune suppression^25^. A high M1/M2 ratio reflects an active cytotoxic and inflammatory microenvironment, while M1 loss and M2 predominance are associated with poor outcomes and response to therapy, including immune checkpoints (ICIs)^26,27^. All together these data fit with our clinical findings showing fewer complete responders among HER2-low compared to HER2-0 TNBC patients treated with pembrolizumab-based regimens. Whether this reflects an inherently lower chemosensitivity of HER2-low tumors, as suggested by some^28^ but disputed by other studies^29^, or is instead a consequence of the specific immune infiltratation associated with the HER2-low phenotype, remains unclear. Importantly, this opens the possibility for future drug combinations. Preclinical studies have already demonstrated that combining ADCs, such as T-DXd, with ICIs promotes immunogenic cell death and increases tumor infiltration by cytotoxic (CD8^+^) T cells^30^. In breast cancer patients, we must await the results of the TRUDI trial, which is currently evaluating T-DXd combined with durvalumab in women and men with HER2-positive and HER2-low inflammatory disease^31^. This study will be instrumental in determining the clinical value of this combination and in clarifying its underlying mechanism of action, as serial tumor samples are being collected longitudinally for correlative analysis.

Our study is the first to show that HER2 expression plays a non-negligible role in depleting the humoral response in HER2-negative breast cancer. Notably, we found that HR-positive tumors are deprived of plasma cells, whereas HR-negative of follicular helper T cells. Tertiary lymphoid structures (TLS) containing germinal center, B cells and follicular helper T have been associated with favorable outcomes in early-stage breast cancer^32^, and more recently with response to immunotherapy in other malignancies^33^ While B cells contribute to TLS formation and adaptive immunity, and their antibodies help recruit NK cells and macrophages to eliminate tumor cells, regulatory B cells may instead facilitate immune evasion^23^. Which of these dynamics dominate in HER2-low tumors remains unclear. Nevertheless, the B-cell compartment emerges as a promising biomarker for patient stratification and a new frontier for therapeutic intervention also in HER2-low breast cancer.

Finally, we found that resting mast cells and neutrophils showed opposite associations with HER2 expression depending on hormone receptor status. Higher HER2 was associated with mast cell enrichment in HR-positive tumors but not in HR-negative tumors, consistent with estrogen-driven pro-angiogenic remodeling^34^^,35^. Conversely, higher HER2 was associated with neutrophils in HR-negative but not in HR-positive tumors, which may be explained by the inherently known role of neutrophils in TNBC^23^. These findings emphasize the need to consider the hormonal context when interpreting impacts on angiogenesis, immune evasion, and therapy response.

This study has several important strengths. First, by evaluating two independent, well-characterized internal cohorts and confirming our observations in an external dataset, we enhanced the consistency of our results. Second, by employing a gene expression-based HER2 classifier that treats HER2 as a continuous variable, we overcame the constraints of categorical IHC and uncovered more nuanced associations between HER2 expression and immune infiltration along the continuum of low HER2 expression. Third, our comprehensive profiling of immune cell population provided a detailed map of the HER2-low immune landscape across different HR contexts, revealing patterns linked to HER2 expression that have not been systematically described before. Still, several limitations must be acknowledged. The retrospective nature of the study precludes causal inferences, although the inclusion of multiple independent cohorts helps mitigate selection bias. Additionally, our estimates of immune cell populations were derived from bulk gene expression deconvolution using CIBERSORTx, lacking spatial or single-cell resolution necessary to characterize the cellular interactions that drive anti-tumor immunity. Finally, our exploratory clinical cohort was small and not powered to detect subtle differences across HER2 subgroups.

In conclusion, the findings of this study deepen our understanding of HER2-low immunobiology and prompt consideration of HER2 heterogeneity as a non-negligible factor for developing immunotherapy in patients with HER2-negative breast cancer. Overall, we provide insights supporting tailored strategies to overcome HER2 expression-associated immunodepletion, including combinations of immunotherapy with ADCs or agents targeting B-cell/TLS axes, aiming to expand options in HER2-negative breast cancer patients.

## Supplementary information

Supplementary material_HER2-low_TIIC

## Data Availability

No datasets were generated or analysed during the current study.
