# Supplementary material for: HER2-low breast cancer is immune-cold: insights into tumor-infiltrating immune cells and implications for immunotherapy
Source: NPJ Breast Cancer. 2025 Dec 3;12:8. doi: 10.1038/s41523-025-00867-z (PMC12796185; doi:10.1038/s41523-025-00867-z)
Supplement: Supplementary file 1 — Supplementary material_HER2-low_TIIC [file 41523_2025_867_MOESM1_ESM.docx]

# **Supplementary material**

# **Supplementary Table 1** HER2 expression (20-gene signature) and tumor-infiltrating immune cell (TIIC) populations (CIBERSORTx)

| **TIICs** | **Discovery cohort** | **Confirmatory cohort** | **External independent (ABiM) cohort** |
| --- | --- | --- | --- |
|  | **ρ (95% CI)** | **ρ (95% CI)** | **ρ (95% CI)** |
| B cells naïve |  | -0.15 (-0.36;0.08) | -0.07 (-0.18;0.04) |
| B cells memory | -0.04 (-0.24;0.17) | p= 0.654 |  |
| Plasma cells | 0.13 (-0.07;0.32) | -0.19 (-0.41;0.04) | -0.26 (-0.36;-0.15) |
| T cells CD8 | -0.01 (-0.23;0.21) | **-0.34 (-0.52;-0.13)** | **-0.30 (-0.40;-0.19)** |
| T cells CD4 naïve | p= 0.513 | p= 0.733 |  |
| T cell CD4 memory | p= 0.062 | p= 0.076 | -0.21 (-0.32;-0.1) |
| T cells CD4 memory activated |  | p= 0.001 |  |
| T cells follicular helper | -0.01 (-0.21;0.18) |  | -0.24 (-0.34;-0.13) |
| T cells regulatory | 0.16 (-0.05;0.35) | 0.15 (-0.07;0.37) | -0.17 (-0.28;-0.06) |
| T cells γδ |  | p= 0.027 | p=0.002 |
| NK cells resting |  | p= 0.699 |  |
| NK cells activated | 0.01 (-0.18;0.21) | 0.15 (-0.07;0.38) | -0.22 (-0.33;-0.11) |
| Monocytes | -0.18 (-0.37;0.03) | p= 0.513 | -0.01 (-0.12;0.10) |
| Macrophages M0 | -0.11 (-0.30;0.09) | 0.02 (-0.21;0.23) | -0.26 (-0.36;-0.16) |
| Macrophages M1 | **-0.32 (-0.49;-0.12)** | **-0.62 (-0.75;-0.46)** | **-0.33 (-0.43;-0.22)** |
| Macrophages M2 | -0.07 (-0.26;0.12) | 0.26 (0.03; 0.47) | 0.08 (-0.04;0.19) |
| DC resting |  | p= 0.617 | p= 0.588 |
| Mast cells resting | -0.04 (-0.23;0.15) | 0.06 (-0.15;0.27) | 0.17 (0.05;0.28) |
| Mast cells activated | p= 0.080 |  |  |
| Neutrophils | p=0.783 |  | p=0.027 |

Bold values, significant findings; Spearman’s rank correlation coefficients (ρ)>0.30; 95% confidence intervals (95%CI) are reported for continuous TIICs; p-values from Wilcoxon tests for dichotomous TIICs; blank cells, TIICs not estimated as reported in the Methods; NK, natural killer; DC, dendric cells.

# **Supplementary Table 2** HER2 expression (20-gene signature) and tumor-infiltrating immune cell (TIIC) populations (CIBERSORTx) by hormone-receptor (HR) status

| **TIICs** | **HR-positive**  (n=255) | **HR-negative**  (n=63) |
| --- | --- | --- |
| B cells Naïve | -0.15 (-0.28;-0.03) | -0.19 (-0.43;0.07) |
| Plasma cells | -0.27 (-0.38;-0.15) | -0.10 (-0.33;0.16) |
| T cells CD8 | -0.25 (-0.37;-0.12) | -0.27 (-0.50;-0.001) |
| T cells CD4 memory | -0.18 (-0.30;-0.04) | -0.16 (-0.40;0.10) |
| T cells follicular helper | -0.04 (-0.16;0.08) | -0.37 (-0.56;-0.13) |
| T cells regulatory | -0.07 (-0.20;0.06) | -0.05 (-0.29;0.18) |
| NK cells activated | -0.19 (-0.31;-0.05) | -0.11 (-0.35;0.14) |
| Monocytes | -0.03 (-0.16;0.09) | -0.08 (-0.31;0.14) |
| Macrophages M0 | -0.11 (-0.23;-0.001) | -0.10 (-0.36;0.17) |
| Macrophages M1 | -0.19 (-0.30;-0.06) | -0.39 (-0.59;-0.15) |
| Macrophages M2 | 0.07 (-0.06;0.20) | 0.04 (-0.22;0.28) |
| Mast cells resting | -0.16 (-0.28;-0.03) | 0.24 (-0.001;0.44) |
| Neutrophils | p= 0.008 | p= 0.003 |
| DC resting | p= 0.129 | p= 0.382 |
| T cells γδ | p= 0.017 | p= 0.531 |

# Spearman’s rank correlation coefficients (ρ); 95% confidence intervals (95%CI) are reported for continuous TIICs; p-values of Wilcoxon tests for dichotomous TIICs; NK, natural killer; DC, dendric cells. Data refer to the ABiM cohort.


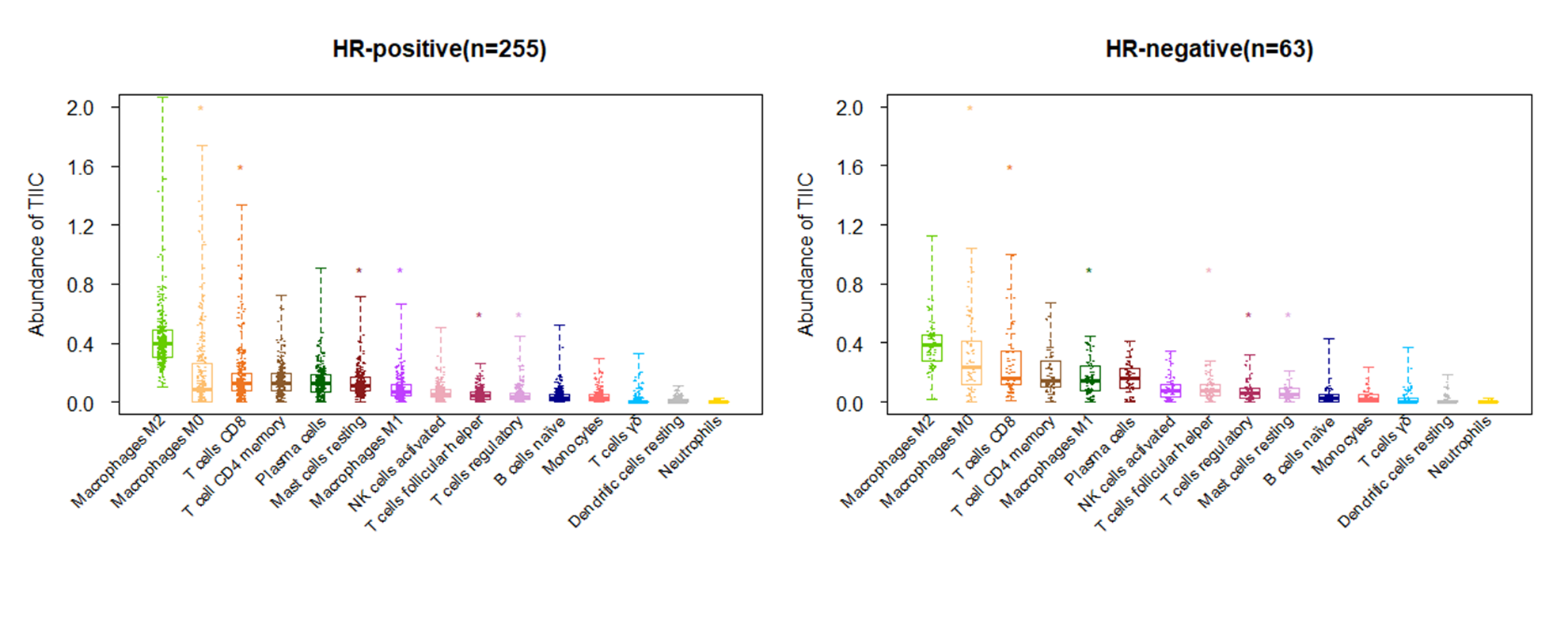
**Supplementary Fig. 1:** Tumor-infiltrating immune cell (TIIC) abundance by hormone receptor (HR) status

**Supplementary Fig. 1 legend**

A total of 15 TIICs were estimated in the ABiM cohort (n= 318). T cells γδ, dendric cells resting and neutrophils were considered as continuous variables. Stars indicate statistically significant differences in TIIC abundance between hormone receptor (HR)-positive and negative (HR-negative) tumors (Bonferroni adjusted p< 0.05).
